# Increased Pupil Size during Future Thinking in a Subject with Retrograde Amnesia

**DOI:** 10.3390/brainsci12010115

**Published:** 2022-01-15

**Authors:** Claire Boutoleau-Bretonnière, Estelle Lamy, Mohamad El Haj

**Affiliations:** 1 CHU de Nantes, Inserm CIC04, 44000 Nantes, France; claire.boutoleau-bretonniere@chu-nantes.fr (C.B.-B.); estelle.lamy@chu-nantes.fr (E.L.); 2 CHU de Nantes, Department of Neurology, Centre Mémoire de Ressources et Recherche, 44000 Nantes, France; 3 Laboratoire de Psychologie des Pays de la Loire, Nantes Université, Univ Angers, 44000 Nantes, France; 4 Unité de Gériatrie, Centre Hospitalier de Tourcoing, 59200 Tourcoing, France; 5 Institut Universitaire de France, 75000 Paris, France

**Keywords:** future thinking, pupil, pupillometry, retrograde amnesia

## Abstract

Recent research has assessed pupil size during past thinking in patients with retrograde amnesia. Building on this research, we assessed pupil size during future thinking in a retrograde amnesia patient. To this end, we measured pupil size during past and future thinking in L, a 19-year-old, right-handed man free of neurological/psychiatric disorders except for retrograde amnesia that occurred after an episode of fugue. During a past thinking condition, we invited L to retrieve retrograde events (i.e., events that occurred before amnesia) and anterograde events (i.e., events that occurred after amnesia). During a future thinking condition, we invited him to imagine events that might occur the following week, the following month, and in the new year. Past and future thinking occurred while L’s pupil size was monitored with eye-tracking glasses. L demonstrated higher specificity during future than during past thinking. Critically, the results demonstrated a larger pupil size during future than during past thinking. The larger pupil size during future thinking observed in L can be attributed to the high cognitive load involved in future thinking. Our study not only demonstrates preserved future thinking in a patient with dissociative retrograde amnesia, but also shows that pupillometry can be used for the physiological assessment of future thinking in retrograde amnesia patients.

## 1. Introduction

Retrograde amnesia refers to failure to remember events that occurred prior to an incident in contrast to the normal or minimally impaired ability to encode and retrieve events occurring after it [1,2]. Retrograde amnesia can be triggered not only by neurological incidents such as traumatic brain injury [3], hypoxia [4], and herpes encephalitis [5], but also by psychological incidents such as extremely stressful experiences (e.g., personal threat, war and military-related activities, natural disasters) or dissociative fugue [2,6,7]. However, the distinction between neurological and psychogenic causes of retrograde amnesia is not always easy, as these causes may be combined [8]. Critically, retrograde amnesia can be associated not only with difficulties in retrieving events encoded before the onset of amnesia, but also with difficulties in future thinking (i.e., in the ability to travel forward in time to imagine or simulate events that may occur in the future) [9]. Considering future thinking in retrograde amnesia, the aim of this paper was twofold. First, we assessed the ability of L, an individual with retrograde amnesia, to construct future events. Second, we assessed whether his future thinking was related with his pupil size. We discuss research assessing pupil size during past and future thinking in normal persons as well as studies assessing pupil size during past thinking in patients with retrograde amnesia.

The pupil is the area of the iris that allows light to enter the eye and reach the retina. Its diameter typically varies from 2 to 4 mm in bright light to 4 to 8 mm in the dark [10]. Two sets of smooth muscles in the iris control the pupil size, namely, the sphincter and the dilator muscles. Whereas the sphincter muscles decrease the diameter of the pupil, the dilator muscles increase it [11,12]. The pupil reacts to three distinct kinds of stimuli: (1) it dilates in response to darkness, (2) it dilates in response to distant fixation, and (3) it dilates in response to increased cognitive effort [13].

Because pupil size increases with cognitive effort [14,15,16,17], a study by El Haj et al. [18] investigated whether it can index cognitive effort during past thinking. To this end, El Haj, Janssen, Gallouj, and Lenoble [18] invited healthy young adults to retrieve personal memories in a past thinking condition and to count aloud in a control condition. In both conditions, the participants faced a white wall so that any variation in pupil size was not attributable to external stimuli, and the pupil size was monitored with eye-tracking glasses. The results showed a larger pupil size during past thinking than during the control task. This increased pupil size was attributed by El Haj, Janssen, Gallouj, and Lenoble [18] to the search and retrieval processes involved in reconstructing memories during past thinking. The effect of cognitive processing on pupil size during past thinking was further demonstrated by Jansse, et al. [19], who monitored pupil size during past thinking in healthy young adults. Pupil size was measured while the participants retrieved personal memories with the help of cue words (i.e., effortless past thinking) or without the help of cue words (i.e., effortful past thinking). The analysis demonstrated less retrieval time and less pupil dilation during effortless than during effortful past thinking. These findings demonstrate how pupil size can be modulated by the cognitive load of past thinking.

Research has also demonstrated how pupil size can index future thinking. This issue was investigated by El Haj and Moustafa [20] who monitored the pupil size of healthy young adults in past and future thinking conditions. In the past thinking condition, the participants retrieved past personal events, while in the future thinking condition, they imagined an event that might occur in the future. The results demonstrated a larger pupil size in the future thinking condition than in the past thinking condition. According to El Haj and Moustafa [20], while past and future thinking require information retrieval from memory, future thinking requires additional cognitive processes such as the ability to recombine the retrieved information into novel scenarios, thus calling upon additional cognitive processing that may increase pupil size during future thinking.

The above-mentioned research [18,19,20] assessed pupil size during past and future thinking in healthy participants. Recently, we assessed pupil size during past thinking in a person with retrograde amnesia [21]. L is a 19-year-old right-handed man without any history of neurological or psychiatric disorders who developed retrograde amnesia following a dissociative fugue. We invited him to retrieve retrograde memories and anterograde memories while his pupil size was monitored with eye-tracking glasses. The results demonstrated impaired retrograde retrieval but successful anterograde retrieval as well as decreased pupil size during retrograde retrieval. This decreased pupil size was attributed to a decline in the cognitive processes involved in retrograde retrieval, i.e., a decline in the processes involved in searching, accessing, and developing memories during retrograde retrieval. While that study was the first to assess pupil size during past thinking in retrograde amnesia, there is still, to our knowledge, no published research on pupil size during future thinking in retrograde amnesia. This is an important issue because retrograde amnesia may involve diminished future thinking. This was demonstrated in retrograde amnesia patients by De Luca, Benuzzi, Bertossi, Braghittoni, di Pellegrino, and Ciaramelli [9], who assessed past and future thinking in a person who developed retrograde amnesia following hypoxia due to cardiac arrest. The person demonstrated difficulties in retrieving retrograde memories during past thinking and difficulties in imagining personal future events during future thinking.

Since retrograde amnesia can be associated with decreased future thinking [9], we assessed whether future thinking in retrograde amnesia patients can be indexed by pupil size. To this end, we extended our previous study [21] in which we measured pupil size during past thinking in L by measuring his pupil size during future thinking. To provide an in-depth assessment of pupil size during future thinking in L, we assessed his pupil size in conditions of near and distant future thinking.

## 2. Method

### 2.1. Case Study

**Figure 1:**
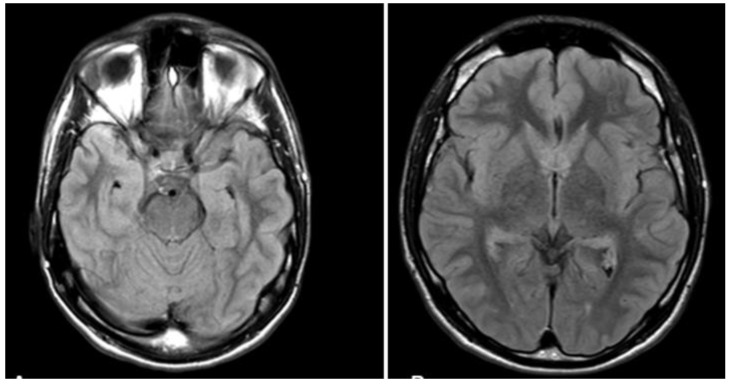
MRI scans of L.

The study involved L, a 19-year-old, right-handed man without any history of psychiatric or neurological disorders. L is in the first year of a BA Law degree. In a dissociative fugue episode on 9 December 2020, L suddenly found himself at the railway station and had no idea about what he was doing there or even about his own name, age, and address. After being questioned by the police at the station, L was taken by the health service to the neurology department of the Nantes Hospital. During the neurological exam, L was conscious and had no neurological/psychiatric symptoms. The medical exam showed normal heart condition and blood pressure. L was also examined with magnetic resonance imaging (MRI) performed with a 1.5-Tesla scanner with Diffusion Tensor Imaging, T1-weighted without and with contrast agent, and 3D T2-SPACE fluid-attenuated inversion recovery (FLAIR) sequences. As illustrated in Figure 1, MRI showed no cerebral abnormality.

Regarding the amnestic symptomatology, the fugue episode left L with a dense retrograde amnesia, observed during the initial and following medical/psychological testing. While L had insight into his amnesia, he was unable to remember specific personal events that occurred prior to the fugue episode. However, after the fugue episode, L re-learned much information about his friends and family. He also managed to attend the university and prepare for his exams.

### 2.2. Procedure

The study was designed in accordance with the Declaration of Helsinki, and L provided his written consent. L was tested in a quiet room at the University of Nantes. Testing included assessments of cognitive functioning and past and future thinking. Unlike past and future thinking testing, cognitive assessment was performed without pupil recording, because pupil size could be influenced by the visual materials used for cognitive testing (e.g., the cube-copying subtest on the Montreal Cognitive Assessment in which pupil activity can be attributed to the perception of the cube rather than to the mental activity involved in copying). These tasks are different from the past and future thinking tasks which involve no visual stimuli, as described below.

### 2.3. Cognitive Testing

We assessed overall cognitive functioning with the Montreal Cognitive Assessment, working memory with the forward and backward spans, verbal fluency with word fluency, inhibition with the Stroop task, and shifting with the Trail Making Test. The score of L on the Montreal Cognitive Assessment was 28 points, i.e., in the normal range. On the forward and backward spans, L was able to retain eight digits and seven digits, respectively. On the verbal fluency test (producing words beginning with the letter P for two minutes), L correctly produced 30 words (percentile 75–90). On the Stroop task, his nomination score (i.e., reaction time) was 43 s (percentile 5), his reading score was 33 s (percentile 10–25), his interference score was 81 s (percentile 25), and he made no perseverative errors. On the Trail Making Test, his shifting score (i.e., reaction time when shifting between letters and numbers) was 22 s (percentile 25), and he made no perseverative errors.

### 2.4. Past and Future Thinking

In the following chronological order, we invited L to retrieve two retrograde events and two anterograde events (i.e., past thinking), as well as to imagine two events occurring the following week, two events occurring the following month, and two events occurring the following year (i.e., future thinking).

During both past and future thinking, L wore eye-tracking-glasses consisting of a remote pupil-tracking system (produced by Pupil Lab) with a gaze position accuracy of < 0.1° and an infrared illumination with a 200 Hz sampling rate. To ensure that differences in retinal illumination would not influence pupil size, the blinds were closed, and the lightness of the room (360-lumens fluorescent lamp) was held constant during testing. During the procedures, L was seated in front of a white wall free of visual stimuli such as drawings, and the distance between him and the wall was approximately 30–50 cm. L was invited not to look away from the wall, but he was free to explore all parts of it. Prior to testing, we calibrated pupil recording by inviting L to fixate a black cross (a 5 × 5 cm cross, printed on an A4 white paper sheet, fixated in the center of the wall). The cross was withdrawn after calibration.

We assessed past thinking regarding retrieval of retrograde and anterograde memories, probing the period before and the period after the amnesic episode, respectively. We invited L to describe personal events (two retrograde + two anterograde events) that he had personally experienced at a specific time and place with as many details as possible. In retrograde retrieval, because L failed to remember any event, we probed retrieval using the cue “school” (i.e., we invited him to retrieve an event which involved school). L replied that he remembered that he was in a primary school near his home without being able to remember further information. To probe further retrieval, we invited him to remember more information about the school. He replied that the school was a couple of minutes’ walk from his home. Despite another round of questioning, L was not able to retrieve any further information. We then tried to probe retrograde memory by inviting L to retrieve an event which involved his family, but he was not able to retrieve any event. Unlike the retrograde condition, L retrieved two anterograde events. The first anterograde event referred to a class the day before at the university that he physically attended with a few classmates, while the majority attended it virtually because of the COVID-19 pandemic. L retrieved details about social distancing in the class, e.g., laughing when seeing a classmate wearing an unusual mask depicting a smiley. The second anterograde event referred to a last-week event with two classmates when they met to study in the library at the university to prepare homework. L retrieved details about his two classmates and how he was able to manage their friendship despite the amnesia. For reasons of confidentiality, we do not provide here all the details related to the events, as is the case for future thinking.

To assess future thinking, we invited L to describe two future personal events that might occur over the coming week. We instructed him to imagine precise and specific events, i.e., events had to last no more than a day, and details had to be provided (e.g., where the event would occur, what he would do, who would be present, what his feelings would be). L described an event involving an exam that would take place the following week at the university and how he would prepare for it. He also imagined details such as the class in which the exam would be held, its duration, and how stressful it would be to finish on time. The other future event referred to a medical exam at the hospital the following week. L imagined himself in the waiting room at the neurological department. He provided details such as the posters on the wall of the waiting room and how he would be anxiously awaiting news about his amnesia.

Besides these two next week events, we invited L to imagine two events that would occur over the following month. L imagined two sporting events in which he would be a player. For both events, L provided details regarding the pitch and the other players, as well as about the trainer who would probably find the players’ performances not up to his standards. We also invited L to imagine events that would occur over the following year. He imagined two trips to two different destinations with his friends. For both events, he provided details regarding what he would do and what he would visit and how he would be happy to discover these places.

### 2.5. Analysis of Specificity

To quantify past and future thinking, we assessed their specificity using a specificity scale in the TEMPau test (Test-Épisodique-de-Mémoire-du-Passé) [22], an instrument derived from classic autobiographical evaluations [23,24] and adapted in French. According to this specificity scale, a past or future event can be evaluated as follows: zero points if there is no retrieved/imagined information or only general information about a theme, one point for a repeated or an extended event, two points for an event situated in time and/or space, three points for a specific event lasting less than 24 h and situated in time and space, and four points for a specific event situated in time and space, enriched with phenomenological details, such as emotion, perceptions, thoughts, or visual imagery.

Based on this specificity scale, and as illustrated in Table 1, we attributed zero points to the retrograde retrieval because L provided only general information about the school, without being able to retrieve any general or specific events and because L retrieved no information regarding the cue “family”. Regarding the two anterograde events, L obtained a mean of four points, as he succeeded in remembering where and when each event had occurred, as well as his own subjective experience during the events (e.g., laughing when seeing his classmate’s mask). Thus, the mean specificity of past thinking of L was (0 + 4)/2 = 2 points. Regarding future thinking, L obtained a mean of four points on the two next week events, a mean of four points on the two next month events, and a mean of four points on the two next year events, as for each event he succeeded in imagining specific events accompanied with spatiotemporal and subjective details. Thus, the mean specificity of future thinking was (4+ 4 + 4)/3 = 4 points. Together, L demonstrated higher specificity of future than of past thinking.

### 2.6. Analysis of Pupil Size

**Figure 2:**
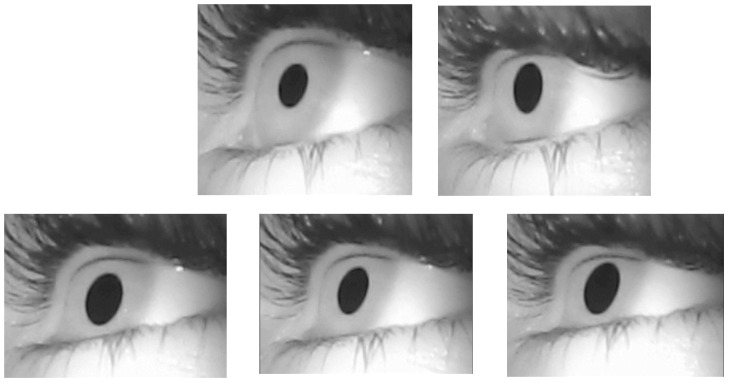
Illustration of L’s pupil diameter during past and future thinking.

We processed the data using Pupil Player software. During data processing, we eliminated blinks and data exceeding typical ranges. In other words, we eliminated data indicating pupil size beyond the interval of 2–4 mm, referring to the typical pupil diameter in bright light, this to exclude 12% of the data. We then calculated the average pupil size (in mm) during each condition, namely, the average pupil size during retrograde retrieval, anterograde retrieval, construction of next week events, construction of next month events, and construction of the next year events. Figure 2 illustrates pupil size in each condition. Because data involved many measures (i.e., each condition involved pupil size measured across hundreds of frames), we were able to conduct statistical analysis for pupil size, as depicted below. This was unlike the specificity score that involved single data for each condition.

### 2.7. Statistical Analysis

We compared pupil size during past thinking (i.e., mean pupil size across retrograde and anterograde retrieval) to pupil size during future thinking (i.e., mean pupil size across the next month, next week, and next year events) using the paired-t test. We then used the paired-t test to compare pupil size during retrograde and anterograde past thinking. Using ANOVA, we also compared pupil size across future thinking (i.e., across the next week, next month, and next year events). For all tests, the level of significance was set at *p* ≤ 0.05, and *p* values between 0.051 and 0.10 were considered as trends, if any.

## 3. Results

### Larger Pupil Size during Future than during Past Thinking

Pupil sizes for each condition are shown in Table 1. Mean pupil size was larger during future (*M* = 2.52, *SD* = 0.41) than during past thinking (*M* = 3.16, *SD* = 0.75), *t*(198) = 7.05, *p* < 0.001, Cohen’s *d* = 1.06. Analysis also demonstrated larger pupil size during anterograde than during retrograde past thinking, *t*(198) = 12.23, *p* < 0.001, Cohen’s *d* = 1.71. No significant differences were observed across the three future thinking conditions, *F*(2, 297) = 1.25, *p* = 0.29, *η*^2^ = 0.031.

## 4. Discussion

In L, a subject with retrograde amnesia, pupil size was larger during future than during past thinking. In addition, and unexpectedly, future thinking was more specific than past thinking.

The higher specificity of future thinking in L contrasts with the study by De Luca, Benuzzi, Bertossi, Braghittoni, di Pellegrino, and Ciaramelli [9], who observed decreased future thinking in their retrograde amnesia case. This apparent contradiction may be explained by a major difference between our case and that of De Luca, Benuzzi, Bertossi, Braghittoni, di Pellegrino, and Ciaramelli [9]. Their case had a cerebral abnormality, i.e., a reduction of gray matter in the thalamus, cerebellum, and fusiform gyrus bilaterally, as well as in regions involved in future thinking, including the hippocampus. On the other hand, L had no cerebral abnormality. Thus, unlike in the case of neurological retrograde amnesia, future thinking may be preserved in the case of dissociative retrograde amnesia, even though some forms of nondeclarative memory can be preserved in cases with hippocampal lesions [25,26]. We suggest that patients with dissociative retrograde amnesia might use their anterograde memories to construct plausible future scenarios. These future scenarios may also involve some retrograde information that is implicitly accessible. This hypothesis is supported by the assumption that dissociative retrograde amnesia can be mediated by suppression processes that serve to keep undesired or distressing memories out of self-awareness or autonoetic consciousness [7,27]. We thus suggest that patients with dissociative retrograde amnesia may avoid distressing retrograde memories by constructing desirable future scenarios. While clinically plausible, our assumption should be considered with caution, because our study does not provide an empirical basis to support it.

Besides demonstrating preserved future thinking in the presence of retrograde amnesia, a key finding of this study is the larger pupil size during future than during past thinking in L, replicating our previous study in healthy participants [20]. This larger pupil size might be due to the cognitive load of future thinking. Although both past and future thinking involve the retrieval of information from memory, future thinking requires more cognitive processes, involved in the flexible recombination of the retrieved information to form novel scenarios [28,29,30]. This assumption is supported by research demonstrating how future thinking is typically more effortful and involves more cognitive processing and reconstruction time than past thinking [31,32]. Thus, the larger pupil size during future than during past thinking, as observed in L, might be due to the higher cognitive load of future thinking with respect to past thinking. This assumption is further supported by the wealth of research demonstrating how pupil size increases with cognitive effort [14,15,16,17]. While cognitive effort can account for the larger pupil size during future than during past thinking in our study, other factors may be involved, such as emotion. It is well known that the pupil typically dilates in response to emotionally laden information compared to neutral information [16,33,34]. This dilation has been observed for a wide variety of stimuli such as emotional video-clips [35] and facial expressions [36,37]. Thus, the larger pupil size during future than during past thinking in our study could be influenced by the emotional load of the constructed future events.

Besides demonstrating a larger pupil size during future than during past thinking in L, we found that pupil size was larger during anterograde than during retrograde retrieval. The decreased pupil size during retrograde retrieval may be due to a decline in the cognitive mechanisms involved in the retrieval of retrograde events, such as the mechanisms involved in the search of information or in accessing it. The decreased pupil size during retrograde retrieval may also mirror a decline in mechanisms involved in the elaboration of memory, if retrieved. It may also mirror failures to use retrieved semantic information as cues to access episodic information. Together, the decreased pupil size during retrograde retrieval can be attributed to a decline in cognitive processing involved in retrograde memory. This assumption is supported by the diminished ability in L to retrieve specific retrograde events, compared with his preserved ability to retrieve specific anterograde events.

Unlike the significant differences between retrograde and anterograde retrieval, we found no significant differences in pupil size between near and distant future thinking. Likewise, there were no significant differences regarding the specificity of near and distant future thinking. Thus, the lack of significant differences in pupil size between near and distant future thinking might mirror the similar specificity of near and distant future thinking. The lack of pupil size differences across future thinking can also be attributed to the possibility that next year events do not necessarily reflect the distant future, unlike events that may occur 5 to 10 years in the future.

Regardless of its potential shortcomings, our study not only demonstrates preserved future thinking in a retrograde amnesia patient, but also offers a clinical perspective regarding the physiological assessment of future thinking in retrograde amnesia patients. We believe that pupillometry has the potential to be used as a practical, non-invasive physiological marker of future thinking in retrograde amnesia patients.

## Figures and Tables

**Table 1:** Specificity scores and pupil size in past and future thinking.

	Past Thinking		Future Thinking		
	**Retrograde** **Events**	**Anterograde** **Events**	**Next Week** **Events**	**Next Month** **Events**	**Next Year** **Events**
**Specificity**	0	4	4	4	4
**Pupil size**	2.06 (0.30)	2.98 (0.69)	3.14 (0.74)	3.08 (0.70)	3.25 (0.78)

Note. A specificity score of four points refers to highly specific events. Pupil size is measured in mm. Standard deviations are given between brackets.

## Data Availability

Data is uploaded.
